# Influence of Preoperative Pain Duration on Microsurgical Varicocelectomy Outcomes

**DOI:** 10.1155/2013/370969

**Published:** 2013-12-26

**Authors:** Mustafa Gökhan Köse, Kadir Önem, Mehmet Çetinkaya, Erkan Karadağ, Emre Arpali

**Affiliations:** ^1^Department of Urology, Baskent University School of Medicine, Ankara, Turkey; ^2^Department of Urology, 19 Mayıs University School of Medicine, Samsun, Turkey; ^3^Department of Urology, Mugla University School of Medicine, Mugla, Turkey; ^4^Department of Urology, Ankara Mevki Military Hospital, Ankara, Turkey

## Abstract

*Objective*. To investigate the question of whether duration of pain before surgery ultimately affects sperm parameters after varicocelectomy. *Methods*. Fifty patients with painful grade-3 varicocele were investigated prospectively. The patients were divided into two groups according to their symptom period. The patients having had grade-3 varicocele for less than 1 year were included in Group-1 (Ge, *n* = 25). Twenty-five patients who had painful grade-3 varicocele for more than 1 year (Gs, *n* = 25) were classified in Group-2. Semen analysis was performed after 3 days of sexual abstinence twice a month. Total sperm concentration (TSC), rapidly progressive motility (SPa), and slow or sluggish motility (SPb) rates were noted. Pain was evaluated by using 10 cm visual analogue scale (VAS). *Results*. Postoperative TSC and %SPb were significantly higher in both groups (*P* = 0.01). There was no difference between two groups for preoperative and postoperative TSC, %SPa, % and SPb values. VAS significantly declined in both groups (*P* = 0.005). This postoperative decline was not significant for intergroup comparison. *Conclusions*. Our results show that increase in semen quality and decrease in the pain after microsurgery varicocelectomy do not depend on the duration of the preoperative pain.

## 1. Introduction

Varicocele is the dilatation and tortuosity of plexus pampiniformis leading to a retrograde reflux. It is estimated in 15–22% of adult male population and 30–40% of men presenting with infertility to the urology clinics [1]. Varicocele is detected on the left in 75–95% of the cases. The etiology of varicocele is controversial. There are some theories such as anatomical differences between left and right testicular veins, the lack of venous valves, and nutcracker phenomenon [2–5].

Varicose veins may cause pain. The etiology of varicocele is similar to peripheral varicose veins. It is not enlightened yet whether varicocele may cause pain because of testicular damage. Our hypothesis is that if the pain was due to testicular damage, the longer duration of pain before surgery would negatively influence varicocelectomy outcomes.

## 2. Materials and Methods

After being approved by the Ankara Mevki Military Hospital Local Ethical Committee (2008), 50 patients with painful grade-3 varicocele were investigated between 2008 and 2012, prospectively. The patients were divided into two groups according to their pain duration. Before randomising patients, one year was accepted as a cut-off value for preoperative pain duration. The patients who had grade-3 painful varicocele for less than one year were included in Group-1 (Ge, *n* = 25) whereas Group-2 (Gs, *n* = 25) included the patients who had grade-3 painful varicocele for more than one year. In the first visit, duration of the symptoms, type of the pain, other scrotal pathology or scrotal surgeries, duration of the pain, sexual partner, and number of children were inquired. The patients who have undergone varicocelectomy previously were excluded to avoid bias. The degree of varicocele was evaluated by physical examination and graded as described in urological literature. Color Doppler testicular ultrasound (CDT USG) was performed to every patient to reveal other scrotal pathology and testis atrophy. Semen analysis was performed after three days of sexual abstinences twice a month. Total sperm concentration (TSC), rapidly progressive motility (SPa), and slow or sluggish motility (SPb) rates were noted. TSC was reported as million/mL. The rates for SPa and SPb were calculated as percents. Pain was evaluated by 10 cm visual analogue scale (VAS). Total testosterone values were only checked in patients who had a TSC of 5 million/mL. The cases had a TSC of 10 million/mL and/or %Spa + SPb < 50%, or severe scrotal pain persistent to conservative methods was encouraged for the surgery. All of the patients were informed about the surgery and informed consent was obtained. Microscopic subinguinal varicocelectomy (under local anesthesia and sedoanalgesia) was performed by the same surgery team. All visible dilated spermatic veins and collaterals were dissected and ligated. Postoperative TSC, SPa, SPb, and VAS were investigated 3 months after the surgery and checked every month in the following visits for four years. The data were taken as the mean and standard error of mean (SEM). Statistical analysis was made by paired samples two test and *P* < 0.05 was accepted as statistically significant.

## 3. Results

The mean of age was 23 ± 3.95 years in Group-1 and 23 ± 2.96 years in Group-2. Duration of pain was 6 (±2.28 SD) months in Ge and 21 (±4.27 SD) months in Gs. All of the cases had left varicocele in Ge. Eight patients had bilateral varicocele in Gs. Two patients in Ge and 1 patient in Gs represented with infertility. None of the patients had atrophic testes. Preoperative and postoperative TSC, SPa, SPb, and VAS values are emphasized in Tables 1 and 2.

For intragroup comparisons, TSC was significantly higher after varicocelectomy (*P* = 0.01). However, when two groups were compared, TSC value did not show difference after surgery. For each group, there was no difference between preoperative and postoperative SPa rates. Increment in SPb after varicocelectomy was statistically different either in Ge or in Gs (*P* < 0.05). On the other hand, it was not different when two groups were compared. VAS declined in two groups after surgery (*P* = 0.005). This decline showed no difference in intergroup assessment.

Total testosterone values were normal in every patient who had a TSC of lower than 5 million/mL. We did not see any recurrence during the follow-up (4 years).

## 4. Discussion 

Varicocele may be accompanied with chronic scrotal pain in 2–14% of the patients [6]. Also 2–10% of the cases who had varicocele suffer from pain [7]. Only the patients who do not respond to conservative therapies such as anti-inflammatory drugs, scrotal elevation, and resting should undergo the surgery. However, when compared with the results of the cases who have undergone varicocelectomy for infertility/subfertility, the overall rate of success in varicocelectomy performed for pain is rather low [8–10].

The major indication for varicocelectomy is infertility. Scrotal discomfort or pain in patients with varicocele is estimated as 2–10% [6]. The pain may be a dull, throbbing pain increasing with exertion and strain [11]. According to Kim et al., urologists meet patients with painful varicocele whose jobs required working in standing positions or vigorous physical effort [12]. They also concluded that microsurgical ligation was an effective treatment of painful varicocele and the quantity and quality of preoperative pain were independent predictive factors of pain resolution after surgery. We agree with Kim in some circumstances. Our patients were all military population who always spend excessive effort and work in standing position. In our study, microsurgical ligation relieved pain in both groups. However, in our trial, the duration of preoperative pain was not significant for the outcome of the surgery.

The results of varicocelectomy recommended in scrotal discomfort or pain are controversial, in the literature. Chen, investigated 76 men with painful varicocele and normal sperm quality [13]. He concluded that the factors predicting symptomatic relief by varicocelectomy in patients with normospermia and painful varicocele nonresponsive to conservative treatment were a greater number of ligated veins (>7), greater preoperative pain score (>6), and longer duration of pain (>9 months). In our study, duration of pain did not influence varicocelectomy results. On the other hand, the limitation for the time of preoperative pain in Chen's study was similar to ours. Our threshold for preoperative discomfort was declared as 1 year to acquire two homogeneous groups. In our study, we searched the effect of preoperative pain duration on varicocelectomy results in oligo- (asthenospermic) patients. This is another different point of our study from Chen's one. However, after we started our study, WHO and EAU revised the criteria for the nomenclature progressive motility (a), nonprogressive motility (b), and nonmotile sperm (c) in 2009. The surgical thresholds were also revised as 40% and 32% for a + b and a. This is a limitation for our trial.

The patients who have palpable varicoceles and abnormal sperm parameters may be encouraged for varicocele repairment. The success rates for painful varicocele were reported as 86–88% for pain resolution; however, the surgical technique is still a dilemma [14, 15]. Armagan et al. investigated the long-term effects of microsurgical varicocelectomy on pain improvement and sperm parameters in patients with varicocele-related pain in 72 patients. They concluded that microsurgical subinguinal varicocelectomy was a reliable approach for clinically varicocele patients with scrotal pain complaints. They showed that regardless of the type of pain, varicocelectomy significantly decreased pain [16].

Altunoluk et al. suggested that subinguinal microscopic varicocelectomy was an effective treatment for painful varicocele. Also, they concluded that the duration of pain preoperatively might predict outcomes [17].

As Armagan et al. conclude, we believe that microsurgical varicocelectomy is a useful method for pain improvement and RECOVERS sperm parameters in patients with varicocele-related pain [16]. However, the main goal of our study is to reveal the influence of symptom duration on the results of varicocele repairment. In this study, we searched the effects of pain duration on varicocelectomy results in oligo- (asthenospermic) patients where Chen only investigated the relief of pain in normospermic patients. Altunoluk et al. recommended microsurgical varicocelectomy in the treatment of painful varicocele. However, unlike their results, we conclude that the duration of pain preoperatively may not predict outcomes.

Few authors have investigated the duration of scrotal pain in varicocele as a predictive factor [18, 19]. In this study we observed the effect of pain duration to varicocelectomy outcomes in patients who had oligospermia. This is the distinctive point of this trial from other studies.

## 5. Conclusions

Microscopic varicocelectomy is useful in patients with scrotal pain, scrotal discomfort, or bad semen quality. Since varicocele is a progressive disease, varicocelectomy may be recommended in cases that have abnormal semen parameters.

We conclude that increase in semen quality and quantity and decrease in the pain after microsurgery do not seem to be related with preoperative duration of pain.

## Figures and Tables

**Table 1:** Preoperative and postoperative values of total sperm concentration (TSC), rapidly progressive motility (SPa), slow or sluggish motility (SPb), and 10 cm visual analogue scale (VAS) in Group-1.

	Preoperative	Postoperative	*P* value
Total sperm concentration (million/mL)	18	34	*P* = 0.01
Rapidly progressive motility (%)	10	11	*P* > 0.05
Slow or sluggish motility (%)	32	43.3	*P* = 0.01
Visual analogue scale (VAS)	5.7	3.5	*P* = 0.005

**Table 2:** Preoperative and postoperative values of total sperm concentration (TSC), rapidly progressive motility (SPa), slow or sluggish motility (SPb), and 10 cm visual analogue scale (VAS) in Group-2.

	Preoperative	Postoperative	*P* value
Total sperm concentration (million/mL)	13	38.8	*P* = 0.01
Rapidly progressive motility (%)	9	10.5	*P* > 0.05
Slow or sluggish motility (%)	34	48.9	*P* = 0.01
Visual analogue scale (VAS)	5.4	3.5	*P* = 0.005
